# Investigating the impact of rice blast disease on the livelihood of the local farmers in greater Mwea region of Kenya

**DOI:** 10.1186/2193-1801-2-308

**Published:** 2013-07-10

**Authors:** Joseph Kihoro, Njoroge J Bosco, Hunja Murage, Elijah Ateka, Daigo Makihara

**Affiliations:** Jomo Kenyatta University of Agriculture and Technology, P.O BOX 62000, Nairobi, 00200 Kenya; Nagoya University, Furo-cho, Chikusa-ku, Nagoya 464-8601 Japan

**Keywords:** Rice blast disease, Livelihood, Socio-economic activity, Geographical distribution, GPS

## Abstract

Rice is the most important cereal crop in Kenya coming third after maize and wheat. It forms a very important diet for a majority of families in Kenya. The demand for rice in Kenya has seen a dramatic increase over the last few years while production has remained low. This is because rice production has been faced by serious constraints notably plant diseases of which the most devastating is rice blast. Rice blast is known to cause approximately 60% -100% yield losses. It is caused by an Ascomycete fungus called *Magnaporthe Oryzae*. The aim of this study was to investigate the impact of rice blast disease on the livelihood of the local farmers in Greater Mwea region and develop a rice blast disease distribution map using GIS approach. The study methodology employed a questionnaire survey which were subjected to sample population of households in the 7 sections with 70 blocks within Mwea region. The collected data was analysed using SAS Version 9.1. Descriptive statistics were used to summarize the household characteristics, the farm characteristics and the farmers’ perceptions of rice blast disease. In the questionnaire, farmers’ response on whether they had been affected by rice blast disease and the total production per acreage was used to develop an attribute table with GPS points. The GPS points were interpolated to create a geographical distribution map of rice blast disease. From the research findings almost all the farmers’ had awareness and knowledge of rice blast disease, 98% of the farmers interviewed were aware of rice blast disease. Out of the 98% with knowledge and awareness 76% have been affected by the disease, while 24% have never been affected. Farmers attributed rice blast disease to a range of different causes, including excessive use of nitrogen fertilizer, water shortage, lack of proper drainage canal and due to climate change. Majority of the farmers interviewed (72%) did not engage themselves in any other socio-economic activity even after being affected by the rice blast disease. 15% opted to growing horticultural crops, 7% engaged in trading activities while 2% started livestock raring, wage earning and Boda boda business.

## Introduction

Agricultural resources are considered to be one of the most important renewable and dynamic natural resources (World Food Programme and Ministry of Disaster Management & Relief 2005). Comprehensive, reliable and timely information on agricultural resources is very much necessary for a country like Kenya, where agriculture is the mainstay of our national economy. But it is being pressurized by high population growth, emergence of new diseases due to climate change and natural hazards like flood, drought and soil erosion. In particular the rice production in Kenya does not meet the food demands for rapidly growing population. Rice farmers in Kenya’s Mwea region are continuing to count losses due to Rice Blast disease. The farmers have been complaining about the disease, which has wiped out almost half of their crop. The disease is still threatening to drastically reduce harvests. An acre of land under rice usually produces on average 25 bags of rice, but this may reduce to 10 bags (Africa Agriculture 2008). Rice blast disease destroyed 5600 hectares (13 840 acres) of rice in Central Province, which produces the bulk of Kenya’s rice. This is equivalent to 10 to 20 percent of annual output and means Kenya will have to increase imports. “This risks worsening Kenya’s food insecurity and makes import of additional quantities even more expensive,” (UN Office for the Coordination of Humanitarian Affairs 2008).

## Materials and methods

### Description of the study site

The Mwea Irrigation Scheme is located in the lower slopes of Mt. Kenya, in Kirinyaga county of Kenya. It is bounded by latitudes 37^0^13′E and 37^0^30′E and longitudes 0^0^32′S and 0^0^46′S. Annual average precipitation for Mwea is 950 mm, with the long rains falling between March and May, while the short rainy period is between October and December.

The scheme traverses three agro-climatic zones, with maximum moisture availability ratios ranging from 0.65 for zone III toward the highland slopes, to 0.50 for the vast area covered by zone IV, and to 0.4 for the semi-arid zone V (. Sombroek *et al*^1982^). Moisture availability zones are based on the ratio of the measured average annual rainfall to the calculated average annual evaporation. The area is generally hot, with average temperatures ranging between 23 and 25°C, having about 10°C difference between the minimum temperatures in June/July and the maximum temperatures in October/March.

The predominant soils of the rice-growing areas of Mwea are vertisols (. Sombroek *et al*^1982^). These are characterized by imperfectly drained clays, very deep, dark gray to black, firm to very firm, and prone to cracking. The most appropriate season for rice cultivation in Mwea is from August to December, when temperatures are opportune for grain filling and with less risk of disease incidence (Mukiama and Mwangi 1989). However, this period is also when the river flows are at their lowest, coinciding with the dry season, further putting a strain on water available for irrigation. Rice production is also complicated by the staggered planting calendar implemented in the scheme (. Ijumba *et al*^1990^) since available water is not enough to reach all farmers during the most opportune season.

### Sampling methodology

Structured questionnaires were conducted to the farmers to collect primary data. These structured questionnaires were conducted face to face with the farmers with a view to establish the impact of rice blast disease to the livelihood of the farmers in the area.

Data collected included household characteristics (age, education and gender of head of household and average family size), farm characteristics (average size of farm, type of land tenure, rice variety cultivated, number of years of rice farming, use of inputs including labour) and farmers’ perceptions on rice blast disease (knowledge of rice blast disease, years since rice blast disease first observed, assets sold due to onset of rice blast disease, cause of rice blast disease and when the disease spreads, whether rice blast disease is changing and the reasons why this might be so, resource use and management due to rice blast disease).

A stratified random sampling approach was employed based on all the units in the sections including the out growers. A total of three hundred and twenty five questionnaires were targeted from the total population of 5,576 household. This number was based on Cochran’s sample size formula for categorical data (. Bartlett *et al*^2001^). A List was obtained from previous JICA survey work with fundamental information; i.e., member’s name, land ownership, area and location of farm (s), and house address. All members were grouped according to their respective sections. The first member was selected and every twenties members were also selected automatically from Mwea Section. The second member and every twenties members were selected from Thiba Section. As such, random selection was carried out to all the sections. However, total number exceeded more than expectation, because some block like H1 has 48 members and 3 were selected for interview. H2 has 67 members and 4 were selected. If selected member was not available next candidate in the list was selected.

However, due to financial constraints and the fact that some respondents were not patient enough to complete all the sections of the questionnaire, the above target could not be realized. A total of three hundred and two questionnaires were fully filled and used for the data analysis and this formed a good representative of the target population.

### Data analysis

The collected data was analysed using SAS Version 9.1. Descriptive statistics analysis of means and frequencies was used to summarize the household characteristics, the farm characteristics and the farmers’ perceptions of rice blast disease. The data was then subjected to a chi square Test of Independence and nonparametric analysis of variance (ANOVA). This was conducted at 5% probability level. From the questionnaire, farmers’ response on whether they had been affected by rice blast disease and the total production per acreage was used to develop an attribute table with GPS points. The GPS points were interpolated to create a geographical distribution map of rice blast disease.

## Results and discussion

### Socio-demographic characteristics of farmer respondents

**Figure 1:**
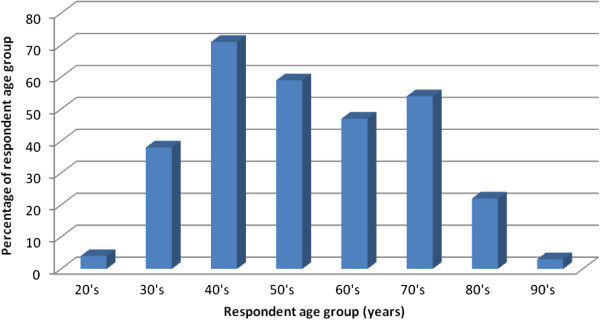
**Age distribution of farmer respondents.**

The sample population of farmer respondents handled during the survey was 302 of whom 19.1% were female while 80.1% were males. The average number of family member is 6.0 per household. Average number of adults above 18 years is 2.19 per household for males, 1.99 for female and 1.79 for children below 18 years. The research study revealed that 47.4% of the respondent had primary level education, 14.9% did not attend school at all, 32.5% had secondary education, while those with Diploma/certificate training and University were 3.6% and 1.7% respectively. The majority of respondent household heads were in the age range of 30’s to 70’s, while none of the respondents was older than 95 years. The age distribution among the sample is shown in Figure 1 below.

### Household economic status

**Figure 2:**
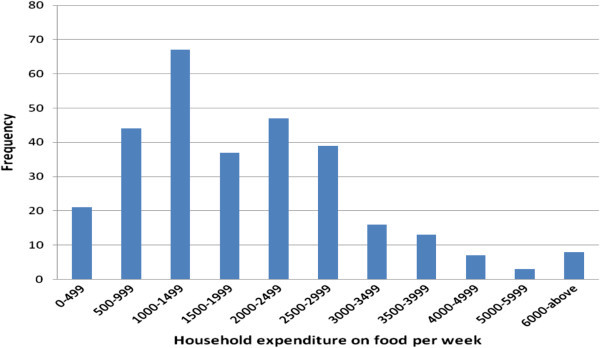
**Food expenditure per person in a week.**

The survey revealed that farming was the mainstay economic activity (73.4% of the respondents) of virtually all the respondents selected for this assessment (Table 1). 12.8% of the respondents are casual labourer, while 7.4% and 3.1% are engaged in business and formal employment respectively. Among them, formal employment has the highest income earning per annum of Ksh118,884 followed by farming with Ksh67,040. 151 farmers have current debts of Ksh36,487.68 on average. The mean weekly expenditure on food is Ksh1,114.60. The distribution of this expenditure is shown in Figure 2. Meanwhile, 228 families on average spent Ksh26,603.07 for education per annum.

**Table 1:** **Average annual income of each social economic activity practiced by the respondent**

Activity	No. of people	% in total adults	Average annual income
Farming	719	73.4	Ksh 67,040
Formal employment	30	3.1	Ksh 118,884
Business	73	7.4	Ksh 55,969
Casual labour	125	12.8	Ksh 39,932
Others	33	3.4	Ksh 41,900

### Land tenure

**Figure 3:**
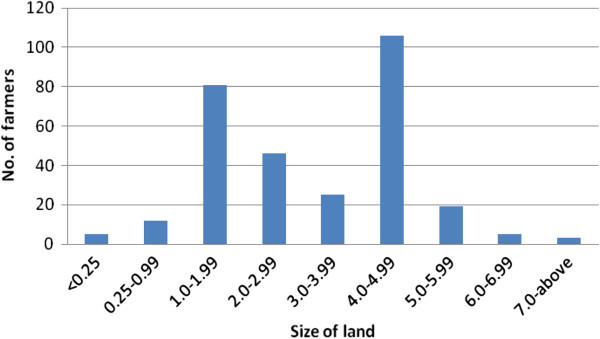
**Land owned in acres for rice cultivation.**

The average land holding is 2.83 acre per household, ranging from 0 to 15.25 acres. However, there are two peaks of the land size ownership, one at about 1.5 acre and the other at 4.5 acres (Figure 3). The common tenure system in the region is land owned but not titled (59%). Most of the farmers were given land in the scheme which was owned by the government through National Irrigation Board (NIB) and the government has not yet issued titles to the farmers up to date. The Land tenure system in the schemes is not favorable to farmers as they do not own land titles making it impossible to access credit. On the other hand women are key players in rice production, but yet they do not own land (NRDS Government of Kenya 2009). 21% of the farmers have title deeds these are the outgrowers who cultivate rice in their own farms around the schemes (Table 2). A bigger number of the farmers 37% received their land as inheritance from their deceased or still living relatives. When asked how much they are willing to pay to rent-in per season, 66% of the farmers were willing to pay Ksh 30,000 per acre in one year while 25% were willing to pay Ksh 35,000 per acre in one year.

**Table 2:** **Land tenure system**

Tenure system	Frequency	Percentage
Title deed	72	21
Owned but not title	207	59
Leasehold	4	1
Government land	13	4
Rented -in	3	1

### Rice production

#### Land preparation and seed source

There are several methods of first land tillage, i.e. Hired tractor private, Hired tractor from National Irrigation Board/Government of Kenya, Use of own tractor, Own oxen, Hired oxen, Family manual labour, Hired labour and farmers Cooperative tractor. From our survey 72% of the farmers uses Hired private tractors in the first land tillage, 18% uses farmers cooperative tractor, 6% uses hired labour while 2% and 1% uses hired tractor from National Irrigation Board and own Oxen respectively. In the second tillage/puddling and leveling animals are mostly used (90%), tractor rotavater 3%, Own labour 4% and Hired labour 3%. Sowing date of the rice seedlings in the field from the nursery is commonly done in July and it continues till August. There is another small peak of sowing in November, and it belongs to the third group of the Irrigation distribution schedule of the Scheme area.

**Figure 4:**
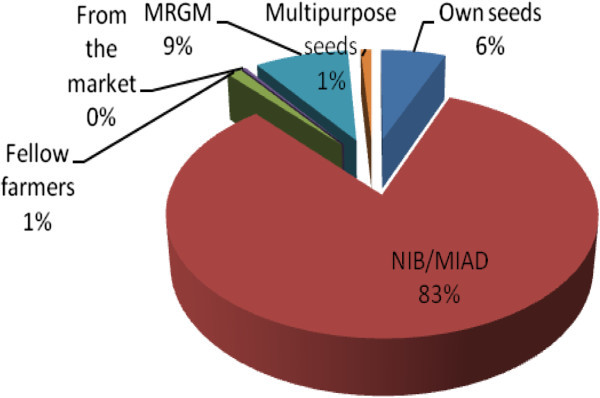
**Farmers source of seeds.**

Although seed source varies, majority of the farmers (83%) source their seeds from Mwea Irrigation and Agriculture Development Centre (MIAD), 9% get rice seeds from Mwea Rice Growers Multi-purpose Co-operative Society (MRGM) a rice grower society in the area who provide seeds on debts and later the farmer pays back in the form of harvested rice. A considerable number (6%) uses their own seeds and very few farmers (1%) obtain their seeds from private seeds companies (Figure 4). The average amount of seeds used in one acre is 22.5 kgs which costs between kshs 80 to Kshs 100 per kg. 97% of the farmers transplant their seedlings randomly and only 3% of the famers transplant their seedlings in line.

### Input use for rice cultivation

Fertilizers are commonly used by the farmers (Table 3). Majority of them use Diammonium Phosphate (DAP) for the basal application and (Sulphur Ammonium) SA for top-dressing. On average one bag (50 kg) of DAP is used for basal application per acre and in a similar way another bag (50 kg) of SA in used for top-dressing in one acre. This signifies that the farmers spend about Kshs 5,000 on fertilizers alone per acre. Organic fertilizers are greatly used during land preparation this reduces the need for a lot of synthetic fertilizers during the growing period. Most of the farmers apply dry (99%) and fresh (97%) animal manure during land preparation. Due to various pests and diseases and foliar application the farmers use kshs740 on average per acre in one cropping season to buy chemicals and foliar fertilizers.

**Table 3:** **Fertilizer use by the farmers in rice cultivation**

Fertilizer use	No.	Kind of fertilizer	Frequency %
Planting fertilizers	1	Diammonium Phosphate (DAP)	87.4
	2	Nitrates, Potash and Potasium (NPK)	4.5
	3	UREA	1.5
	4	Sulphur Ammonium (SA)	3
	5	Muriate of Potash (MOP)	3.6
Top dressing fertilizers	1	Diammonium Phosphate (DAP)	4
	2	Nitrates, Potash and Potasium (NPK)	2.5
	3	UREA	1.4
	4	Sulphur Ammonium (SA)	89.9
	5	MOP/CAN	2.2

### Family and hired labour use for rice production

Farmers in Mwea region highly depend on hired labour in rice cultivation. Birds scaring is the most expensive activity because it has a constant cost, whether you have 2 acres or half an acre the farmers have to employ a person to scare birds for about one and half months before the harvesting. Planting/sowing also requires a lot of money due to the intensive labour requirements. In summary the total family labour, hired labour and mechanization expenditure in one acre is Kshs. 32,494 (Table 4).

**Table 4:** **Average expenditure on family labour, hired labour and mechanization costs for rice production per acre for the main crop in one season**

Rice production	Activity	Costs per acre (Ksh)
Land preparation	Clearing field	1,429
	Repairing Bunds	585
	Repairing canals	649
	1st ploughing	3,466
	2nd ploughing	1,211
	1st harrowing	1,590
	2nd harrowing	1,790
Labour use	Planting/sowing	4,060
	Soil covering	1,133
	1st weeding	1,782
	2nd weeding	1,725
	water management	1,235
	Scaring birds	5,062
	Harvesting	3,632
	Post harvest activities	2,012
Agricultural materials	Fertilizer and chemicals application	495
	Other expenses	638
**Total**		**32,494**

### Harvest and sales of rice

The average yield per acre of basmati variety is 21.7 bags (1,953 kgs) and BW196 variety is 26.03 bags (2,343 kgs), IR2793-80-1 variety did not give a good picture because very few farmers grow it in very small portions of land (Table 5). Some farmers (35%) were not satisfied by the yields they got and a bigger percentage 51% were contented and said that the yields were average. Only 14% of the farmers interviewed were of the opinion that their yields were above average. One acre of rice can produce about 30 bags (2,700 kgs) if proper practices are adhered to.

**Table 5:** **Average harvesting and sale of rice in 2010 cropping season**

Variety planted	Average harvest per acre 90 kg bags	Percentage	Percentage	Average unit price per 90 Kg bag
		Amount consumed	Amount sold	
Basmati370	21.7	12.3	87.7	Ksh 4,473
IR2793-80-1	3.1	76.5	23.5	Ksh 2,500
BW196	26.03	62.4	37.6	Ksh 3,500

**Figure 5:**
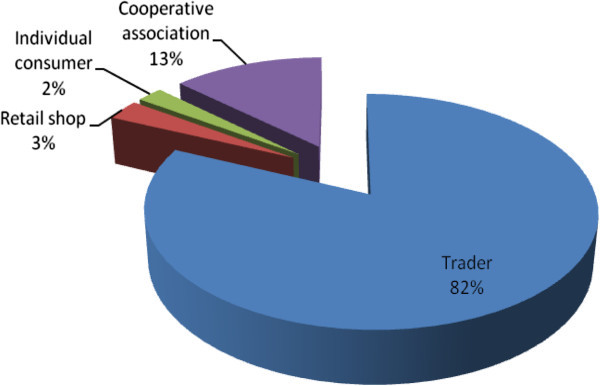
**Farmers marketing channels.**

Basmati variety is generally a cash crop in the region, out of the total harvested rice 87.7% is sold and the rest 12.3% is left for consumption. BW196 is usually grown for consumption, the farmers indicated that BW196 is very heavy and provide a lot of energy compared to basmati but due to lack of aroma and poor cooking qualities most of the people especially in urban area do not like it. 62.43% of BW196 produced is kept for consumption and only 26.03% is usually for commercial purposes. Interestingly, much of the BW196 is sold to the local farmers who do not cultivate the variety (Table 5). In the year 2010, the average sale per bag of basmati variety was ksh 4,473 while that of BW196 was 3,500 this clearly indicate the reason for the choice of basmati over BW196 variety (Table 5). Majority of the farmers 82% sale their rice to traders and 13% sale their rice to cooperative association in the area (Figure 5). The farmers either take their rice to the market or traders come to their gate.

### Profit calculation

Profit in rice growing was obtained by subtracting the average expenditure of rice growing from the average sales of rice produced in one acre. The average expenditure included the family labour, hired labour, mechanization costs and all farm inputs i.e. seeds, fertilizer, chemicals and foliar fertilizers. The total sales were obtained by multiplying the average yield per acre of each variety by the average unit price per 90 kgs bag. The profitability of the two common varieties is as shown in Table 6. Farmers indicated that in cultivating the two varieties the expenditure is almost the same but there is a little difference because the variety BW196 requires more fertilizer than the basmati variety and basmati requires more chemicals. In our average profit calculation we assumed that the average expenditure is similar to the two varieties. From the table below we can say that though BW196 is more productive than basmati it has less returns. The difference in profitability is ksh 5,959 per acre, we also noted that the market demand for basmati is very high compared to BW196 this clearly indicate the reason why 98% of the farmers in mwea region grow basmati rice.

**Table 6:** **Average profitability in rice growing for the year 2010**

Variety	Average sales (Ksh)	Average expenditure (Ksh)	Average profit (Ksh)
Basmati	97,064	40,259	56,805
BW196	91,105	40,259	50,846

### Farmers’ perception on rice blast disease

From the research findings almost all the farmers’ had awareness and knowledge of rice blast disease, 98% of the farmers interviewed were aware of rice blast disease. Out of the 98% with knowledge and awareness 76% have been affected by the disease, while 24% have never been affected. A chi square Test of Independence was performed to examine the relation between farmers’ knowledge on rice blast and rice blast affection. The relation between these variables was significant, *X*^*2*^ (1, *N* = 290) = 6.05, *p* =.014. Farmers’ with knowledge and awareness were less likely to be affected by the rice blast disease than farmers without the disease knowledge and awareness.

Different local names of the disease were identified but the majority of the farmers 93% still refer the disease as blast, other names were kivuruto and baa. Farmers attributed rice blast disease to a range of different causes, including excessive use of nitrogen fertilizer, water shortage, lack of proper drainage canal and due to climate change. The disease results in yield loss as high as 70–80% when predisposition factors (high mean temperature, relative humidity higher than 85–89%, presence of dew, drought stress and excessive nitrogen fertilization) favor epidemic development (. Piotti *et al*^2005^). Farmers’ knowledge on the type of blast in their farm units was diverse, 52% of the farmers find leaf blast, 42% panicle blast, while 6% and 2% observe neck and stem blast respectively. The way the farmers are able to identify the above mentioned type of blast varied widely, some of the common answer were; reddish brown spots on leaves, empty panicles, whitish panicle, yellow leaves, black necks and majority indicated that extension workers from MIAD indentified the type of blast in their farm units. The fungus *Pyricularia oryzae* attacks at all stages of the crop and symptoms appear on leaves and nodes ( See bold *et al.*^2004^).The symptoms are more severe in case of neck blast that is characterized by the infection at the panicle base and its rottening ( Bonman *et al.*^1989^).

**Figure 6:**
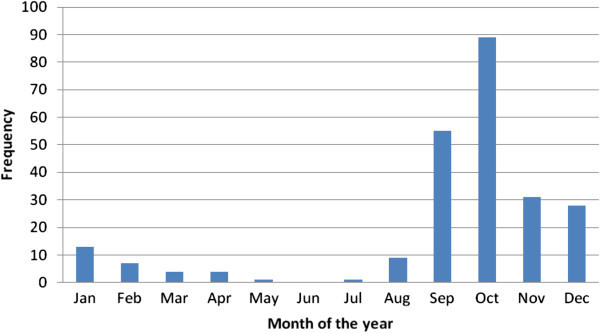
**The month of the year rice blast disease is prevalent.**

The interviews also revealed that rice blast disease is the most destructive disease compared to other diseases the farmers mentioned that it is possible to harvest nothing when affected by the disease. Surprisingly 76% of the farmers have never observed any other disease in their farm while 24% indicated they have been affected by rice blight, leaf minor, stem rot and leaf rust. According to ( Shahijahan *et al.*^2010^) paddy blast is generally considered as the principal disease of rice. 87% of the farmers were first affected by rice blast in the year 2009 and 7% were first affected by the disease in the year 2010 the rest had realized the disease in the year 2003 to 2008. From the findings the month of October has a high disease prevalence compared to the other months (Figure 6).

Rice blast disease seems to be a new disease in Mwea region as indicated early. The effect of the disease in production has become a concern to the region and also to the nation which relay on this region for production of rice to its population. In this research we observed the progression of rice blast disease in the farm units since the year 2006 to 2010 and its effect to the total production per acre. It was noted that during the year 2009 when rice blast occurrence was at 55.5% the average bags (90 kg) that were produced per acre dropped to 10.5 from 21.9 produced in the previous year. This indicated that the total loss in production due to rice blast disease in 2009 was 47.9% compared to the previous year (Table 7). The year 2010 rice blast disease occurrence dropped to 6.2% and the average production per acre went back to normal. Heavy yield losses have been reported in many rice growing countries. For example 75, 50 and 40 percent grain loss may occur in India (Padmanabhan 1965a), Philippines (Ou 1985) and Nigeria (Awodera and Esuruoso 1974). In rice-growing areas, a blast outbreak could cause the loss of about 35–50% of rice yield, and in a serious outbreak of the disease, up to 100% of yield could be lost (WARDA 1999).

**Table 7:** **The percentage rice blast occurrence and the average production in an acre**

Year	% rice blast occurrence	Bags (90 kg) produced in an acre
2006	6.8	22.9
2007	6.3	22.2
2008	9.7	21.9
2009	55.5	10.5
2010	6.2	22.4

**Figure 7:**
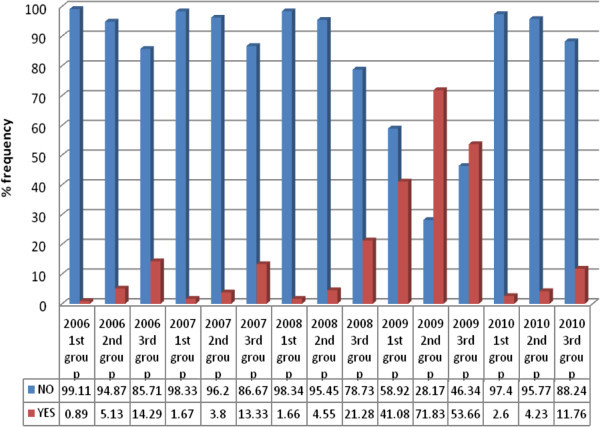
**Percentage rice blast incidences in the various planting group from 2006 to 2010.**

Farmers in Mwea region have three different planting groups. According to the farmers the grouping is done due to the shortage of water. To determine which group you will be located depend on the farm unit you are in and when you pay the water charges. It was educative to see how rice blast disease progresses from 2006 to 2010 in various planting groups. It emerged that in the year 2006, 2007, 2008 and 2010 the farmers in the third planting group were more affected by rice blast followed by farmers in planting groups two and least affected are farmers in planting group one. In 2009, farmers in planting group two were more affected followed by farmers in planting group three (Figure 7).

Various rice varieties are usually susceptible to rice blast disease from this study 97% of the farmers interviewed indicated that Basmati370 is highly susceptible to rice blast disease. Important to note is that BW196 according to the survey is resistant to the disease only 2% of the respondents were of the opinion that the variety is susceptible. In our earlier discussion we saw that farmers mostly look at the variety that will fetch good market prices and thus they better grow Basmati370 that is highly susceptible to the disease than grow a resistant variety like BW196. In China the incorporation of resistance genes, Rice Blast is no longer a serious problem for the widely grown hybrid indica Rice. However, it has remained a serious problem for glutinous Rice (32% losses), japonica Rice (5-12% losses) and upland Rice (losses could reach to 20-50%) ( Youyong *et al.*^2000^).

### Control strategies used by farmers against rice blast disease and factors influencing them

A range of different methods had been tried by the respondents in their attempts to control rice blast disease and some of these may well have made things better. These included: Burning diseased-straw and stubble (3%), Chemical use (82%), Abandon field (1%), Split applications of nitrogenous fertilizer and use of resistance varieties had less than 1% (Table 8). However, by the time of the surveys, the majority of rice farmers (86%) had abandoned attempts at controlling rice blast disease using the aforementioned methods because they were found to be ineffective. A few (4%) indicated that the control methods were too expensive and labourious given the rate of infection of the rice, and households did not have enough labour to carry them out. Farmers considered that hired labour was too expensive. Only 8% of the farmers who were using chemicals thought the method worked very well. In China farmers growing susceptible varieties use fungicide to control Blast, making as many as three to eight spray applications per season (Li Jiarui 1994). The use of resistant varieties is the most economic and effective way of controlling rice blast, especially in resource-poor farmers’ fields ( Séré *et al.*^2011^). Therefore considerable effort should be directed toward developing and identifying blast-resistant cultivars in order to provide farmers with low-cost blast management.

**Table 8:** **Percentage in use of various rice blast control method**

Control method	Frequency	Percentage
Burning diseased-straw and stubble	8	3
Use of resistance varieties	1	0
Chemical use	218	82
Apply compost	0	0
Avoid farm activities when plants are wet	0	0
Abandon field	2	1
Split applications of nitrogenous fertilizer	1	0
Others (Not using any control method)	37	14

A chi square Test of Independence was used to analyze the data with rice blast disease infection as one variable and the control methods as the second variable. There was a significant effect, *X*^2^ (5, *N*= 299) =202.32, *p* = .001. Whether to control or not was influenced by the education of the farmer and the current income from rice. Farmers with a higher income from rice were more likely to attempt methods of controlling rice blast disease than farmers earning a lower income from rice. Surprisingly, however, farmers with higher levels of education were less likely to control the disease than farmers with lower levels of education (*p* < 0.01). This is perhaps because higher education is associated with greater opportunities for generating alternative sources of income, and as such, those farmers who are more highly educated, may have opted to diversify to other sources of income rather than attempt to control rice blast disease.

Most of the farmers surveyed obtained information on control strategies either from extension workers 50%, fellow farmers 23% or from training worker 20%. A smaller number received information from visiting researchers 3% and from the local leaders 2% (Table 9). This indicated that farmers much prefer to get their information through some form of personal contact. Kenya Agricultural Research Institute (KARI) has focused on rice research while the Ministry of Agriculture is providing extension. KARI and its partners have the capacity to conduct rice adaptability trials. The scientists based at research institutions have experience in rice breeding, agronomy, crop protection and socio-economics (xNRDS Government of Kenya 2009).

**Table 9:** **Farmers source of advice on the appropriate method of rice blast disease control**

Source of advice	Frequency	Percentage
Fellow farmers	54	23
Extension workers	119	50
Training workers	47	20
Radio	0	0
Local leaders	5	2
Visiting researchers	7	3
Newspaper/pamphlet	0	0
Others	4	2

From the analysis the brand names of chemicals being used by the farmers are; Topsin, Goldazim, Rodazim and Bavastin. These chemicals are readily available in the market and most of the extension officers from MIAD/NIB train the farmers on how to use these chemicals. In 2009 when the area was highly affected by the disease the government through NIB provided some of these fungicides to the farmers for free. It again emerged that MIAD/NIB are the main source of advice to the farmers on the products to use.

It was surprisingly to note that 51% of the farmers interviewed were of the opinion that no group/institution is carrying out any developmental activity in tackling rice blast disease. 49% of the farmers with the knowledge of a group/institution indicated that MIAD/NIB lead the list with 93% in trying to tackle the disease, Ministry of agriculture followed with 4%, farmers group with 3% while KARI, Agrovets and MRGM with 1% each.

Majority of the farmers interviewed (72%) did not engage themselves in any other socio-economic activity even after being affected by the rice blast disease. 15% opted to growing horticultural crops, 7% engaged in trading activities while 2% started livestock raring, wage earning and Boda boda business (Table 10).

**Table 10:** **Other socio-economic activities introduced as a result of rice blast disease**

Activity	No. of farmers	Percentage
No activities introduced	216	72
Growing other horticultural crops	44	15
Livestock rearing	7	2
Boda boda business	5	2
Trade	22	7
Wage earner	5	2

Due to the loss of produces by the rice blast disease 74% of the farmers liquidated their assets to meet other needs (Table 11). 37% of those farmers liquidated their assets to cater for school fees, 34% for domestic use e.g. purchasing of food stuff, 22% for buying farm inputs for the next planting seasons, 4% for paying debts/loans obtained to facilitate farming activities, while only one farmer liquidated her assets to start a grocery business in Mwea town. A chi-square test was performed and no relationship was found between economic status of the famers and the rice blast disease infection, *X*^*2*^ (2, *N* = 302) = 0.89, *p* =.64.

**Table 11:** **Assets type and value per year liquidated due to the effect of rice blast disease**

No.	Asset type	No. of farmers	Value per annum (Ksh)
1	Land	4	280,000
2	Tractor	1	800,000
3	Motor vehicle	2	220,000
4	Motor cycle	1	50,000
5	Ox cart	2	20,000
6	Ox plough	1	3,000
7	Livestock	71	20,650
8	Trees	1	1,000

### Rice blast disease mapping

During the survey the farmers were spatially sampled within the study area. The sample size was geographically representative of the study area. The farmers were asked whether they had been affected by rice blast disease (Table 12) and the total production per acreage. The rice blast effect on production for the year 2009 was used to produce the density map of rice blast disease in Mwea region. The reason for the use of year 2009 is because rice blast disease was highly recorded in the area and was the main contribute to yield loss.

**Table 12:** **Farm units referenced by GPS through field**

Rice blast occurrence in the farm units	No. of farmers	Percentage
Have been affected by rice blast	226	76.09
Have never been affected by rice blast	71	23.91

**Figure 8:**
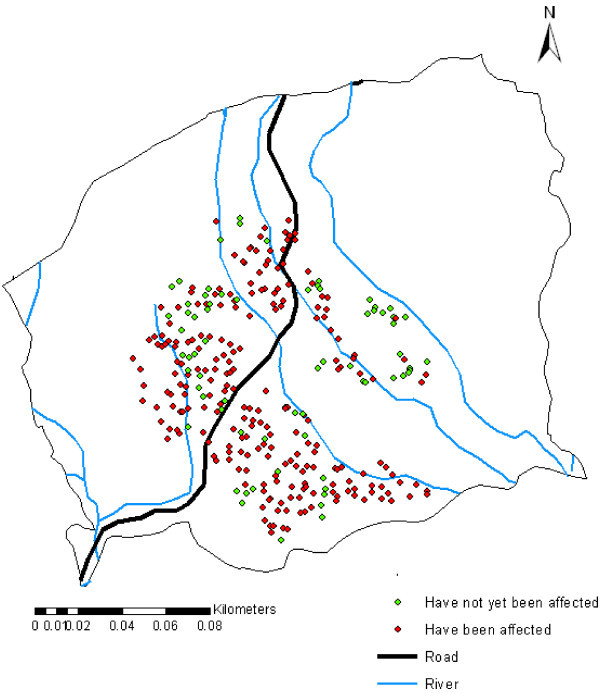
**GPS points displayed on the Mwea map showing cases of rice blast disease on the sampled farm units.**

Figure 8 shows the rice blast disease cases created and pointed with point symbol on the Mwea region map.

**Figure 9:**
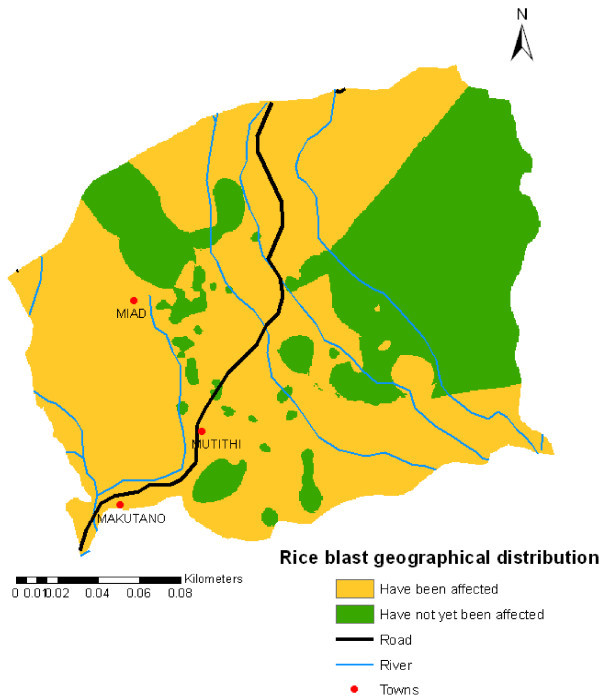
**Geographical distribution of rice blast disease in Mwea region.**

The GPS points were interpolated to create a geographical distribution map of rice blast disease (Figure 9). Interpolation is a way to make a Scientific Wild Ass Guess (SWAG) and is common in biological studies and in studies of disease where samples are infrequent and randomly placed. A simple interpolation method called Inverse Distance Weighting (IDW) was applied. The “inverse” part comes from the first law of Geography; more distant things are less likely to be related than close things. IDW estimates cells value by averaging the values of sample data points in the vicinity of each cell. The closer a point is to the center of the cell being estimated, the more influenced, or weight, it has in the averaging process. This method assumes that the variable being mapped decreases in influence with distance from its sampled location.

**Figure 10:**
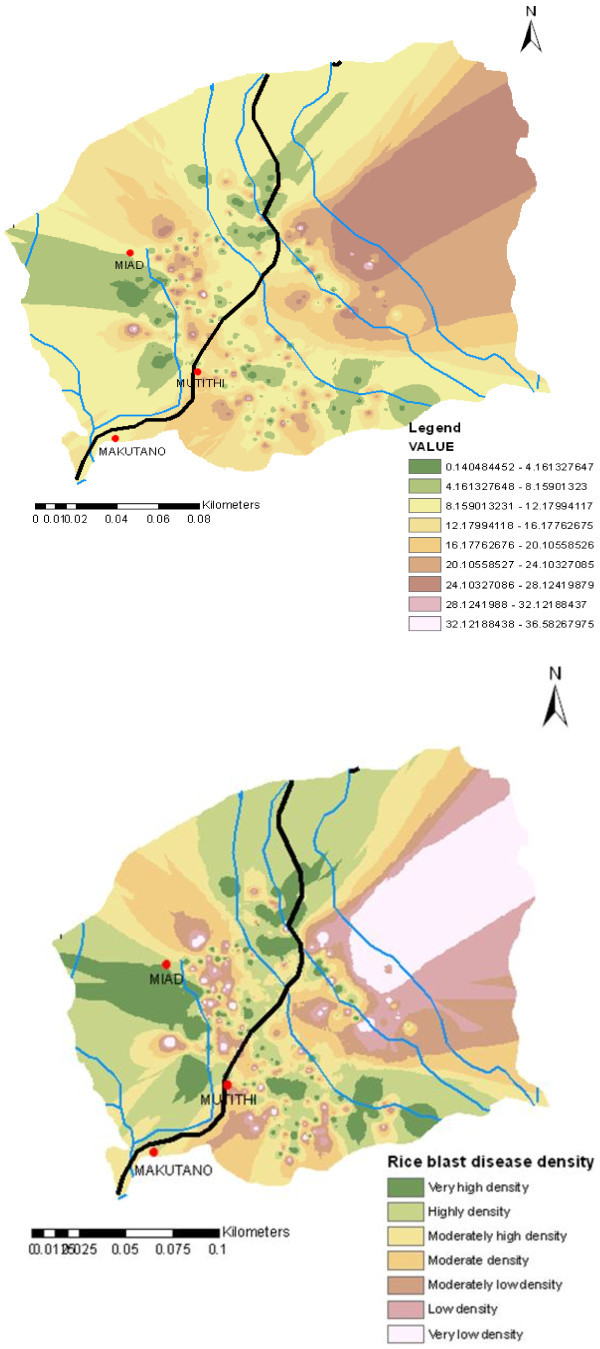
**Rice blast disease density map.**

To create the density map of rice blast disease within Mwea region the production per acre by the farmers was linked to the GPS points and interpolated using ArcGIS 10. In this analysis an assumption was made that the rate of production per acre is directly influenced by the intensity of rice blast disease. An objective scale was developed, this scale was used to measure the preference from worst to best which was based on rice production in the farm units. Having the information from our survey analysis that an average production per acre is normally 21.7 bags (90 kg) a disease density scale was developed as shown in (Table 13). The scale was used to reclassify the interpolated map and a disease density map was created (Figure 10).

**Table 13:** **Rice blast disease density scale as per total acreage production**

Rice production in 90 kgs bags per acre	Rice blast disease density scale
0-4	Very high density
4.1-8.0	High density
8.1-12.0	Moderately high density
12.1-16.0	High density
16.1-20.0	Moderately low density
20.1-24.0	Low density
24.1- above	Very low density

### Conclusion and recommendations

The study demonstrated that rice blast disease had a negative effect on the livelihood of the people in Mwea region. The farmers indicated that in some seasons they harvested nothing due to rice blast disease. This led to the disposal of some of their assets to meet their needs and even some famers stopped rice growing to other activities like trade and growing horticultural produce which in their opinion may had better returns. This greatly affected their socio-economic status and even some of the farmers had to look for loans to buy farm inputs for the next season. Farmers those were economically well up had an upper hand in controlling the disease because they were in a position to acquire the fungicides and reduce the infection. Government through the National Irrigation Board should come up with ways of helping the farmers in preventing and controlling the disease. The disease seems to affect every farmer equally despite their level of education or age.

Rice blast disease was lanked the most destructive disease compared to all the other rice diseases in the region. According to the farmers the main cause of the disease was the use of excessive nitrogen fertilizer. In a study conducted in Suakoko, Liberia, with 16 rice cultivar, the incidence of the blast increased when nitrogen was increased from 60 kg N to 120 kg N ha^-1^ (Awoderu 1983). This indicates the risk of excessive use of nitrogen fertilizers. This is due probably to the injurious effect of ammonium accumulation in the cells of the plants treated with high nitrogen (Ou 1985). The soluble nitrogen in the plants may serve as suitable nutrients for fungus growth. Therefore, to minimize its impact on the rice blast disease, more research is needed to establish an effective level of nitrogen fertilizer in the management of the rice blast disease.

This study concluded that GIS technology has been proven efficient in data collection and presentation of disease incidence for charting immediate corrective and preventive actions. Geographical Information Systems for disease surveillance play a major role in disease mapping. A GIS can clearly provide spatial analytical capabilities to interrogate the data. In order to utilise GIS for these purposes it is important to have clear data collection protocols in place ahead of time, and an awareness of the technical and legal issues around storing and managing such information. The usefulness of GIS to disease mapping are largely dependent on the availability of good quality case data, and any enhancements to the way such information is collected would ultimately enhance the application of the spatial analytics used to assist in disease mapping

In conclusion the current emphasis on rice blast disease should be how to control the disease. Therefore, emphasis should be in developing rice cultivars with adequate levels of resistant/tolerance to the disease. Sound crop management practices would also go a long way to minimize the losses caused by the pathogen in Mwea region.

Future directions for rice blast research and control in a hitherto low to moderate input production system in Mwea should focus on development of sound and practical integrated management programs for the disease and studying the effect of changing cropping practices on disease incidence/intensity (Fomba and Taylor 1994). More to this we need to conduct more studies on the genetics resistance and collate of farmers’ indigenous knowledge and skills in the management of rice blast and other disease/pests.

## Abbreviations

CAN: Calcium ammonium nitrate
DAP: Diammonium phosphate
GIS: Geographic information system
GPS: Global positioning system
IDW: Inverse distance weighted
IWUA: Irrigation water users association
KARI: Kenya agricultural research institute
MOP: Muriate of potash
MRGM: Mwea rice growers multi-purpose co-operative society
NIB: National irrigation board
NPK: Nitrates, potash and potasium
SWAG: Scientific wild ass guess
SAS: Statistical analysis software
SA: Sulphur ammonium
